# Parental emotionality and power relations in heritage language maintenance: experiences of Chinese and African immigrant families in Australia

**DOI:** 10.3389/fpsyg.2023.1076418

**Published:** 2023-06-30

**Authors:** Yining Wang, Vera Williams Tetteh, Sithembinkosi Dube

**Affiliations:** Department of Linguistics, Macquarie University, Sydney, NSW, Australia

**Keywords:** parental emotionality, language ideology, power relations, heritage language, Chinese migrants, African migrants

## Abstract

Emotionality is increasingly given prominence in the field of language acquisition and socialization in migration contexts. This cross-sectional study explores the emotional experiences of Chinese and African immigrant families in their practices of maintaining their children's heritage languages. We used open-ended interviews, field notes from informal conversations and observations, photographic evidence of children's literacy practices, and language portrait (LP) descriptions, to collect data. Results from an ethnographic analysis of the data revealed a whole range of negative and positive parental emotions (e.g., anxiety, loss, shame vs. enjoyment, accomplishment, and pride), in the discourse of maintaining heritage and minority languages. We discuss the language emotions, whether positive or negative, in light of language ideologies, which specifically points to the significance of profit discourse in the formation of family language policies (FLPs). This materialistic valorization reveals the complexities of power relations between English and minority languages, between Chinese and African languages, and within various Chinese and African languages. Consequently, the distinct hierarchies between English and minority languages and the hidden layers within minority languages further legitimate diasporic ideologies of Chinese and African parents in terms of the emotionality associated with prioritizing, maintaining, and forgoing languages. These findings suggests that language emotionality is of vital importance to the psycho-social wellbeing of immigrant families and has practical implications for policymakers and heritage language research.

## 1. Introduction

Yeah into this big dream because if you ask, every parent wants their child to learn Shona or Ndebele. But to actually do it practically, it comes down to[sic] ah to our weak economy. The background that we are coming from […] We are not just working for ourselves, we are working to earn money for ourselves to build our lives but we are also looking after a thousand people that we have left there. So our time with our kids as they grow up to actually nurture them language-wise is very very limited compared to other people. A Chinese person coming here does not have that. They can stay home with their kids, sometimes they will wait until [sic]kids go to junior school even year 5. Or they work normal shifts and just go home without having to do any of that. (Bandi)

Parents such as Lisa and Bandi and their husbands Mandla and Victor migrated from Zimbabwe to seek economic empowerment in Australia. For them, fulfilling obligations with work and/or study as key factors for their migration and living in Australia meant that they were left with limited time to spend with their children and nurture their development in their heritage language. Similar to many other minority immigrant families (e.g., Borland, 2006; Et-Bozkurt and Yagmur, 2022; Romanowski, 2022), the African parents demonstrated a strong desire for passing on their heritage languages to the next generation, but they felt anxious when perceiving that their “big dream” (as voiced by Bandi above) of language nurturing often became stuck due to economic pressures and constraints in the migration context. In fact, emotions, such as desire and anxiety, reveal that language maintenance is not merely a linguistic decision on whether or not to learn a heritage language but is deeply situated within the socio-economic and cultural backgrounds of individual families (Nyarko, 2014) or ethnicities. In explaining how the survival crisis and family burden disadvantaged them from raising their children in relation to African heritage languages (e.g., Shona or Ndebele), Bandi, as shown in the above quotation, made a comparison with Chinese immigrants whose migration and economic situation were perceived to be more advantageous to Chinese heritage language maintenance.

The umbrella term “Chinese” consists of seven major varieties or dialects: Mandarin (the northern), Yue (includes Cantonese), Wu (includes Shanghainese), Kejia [Hakka], Min [Hokkien], Xiang, and Gan, and many of the dialects are mutually unintelligible (Taylor and Taylor, 2014). Despite the varieties of Chinese languages, only one writing system (Chinese characters) is used in China, and Mandarin is the corresponding spoken form of this written standard (Shen and Jiang, 2023). As the official language of the Chinese government and the medium of instruction in schools, Mandarin has taken precedence over all other varieties and enjoys a unique position of prestige in China (Shen and Jiang, 2023). Accompanied by the rise of China's economic and political clout in global affairs, Mandarin has replaced Cantonese to become the new lingua franca in the broader Chinese diaspora, such as in the UK (Curdt-Christiansen and Huang, 2021), Singapore (Tupas, 2015), Ireland (Liu, 2022), and Australia (Wang, 2020). Largely due to the prestigious position of Mandarin, many Chinese, including all the Chinese participants in the current research, habitually use the term “the Chinese language” as the referent of “Mandarin” Chinese. Thus, unless otherwise specified, the phrase “the Chinese language” mentioned in the excerpts often refers to Mandarin. Given China's fast economic growth in the twenty-first century and Australian immigration policy with orientation on economic and skill criteria, these recent Chinese immigrants in Australia represent a group of middle-/upper-class Chinese who are highly skilled, highly educated, and in the high-income bracket (Gao, 2015; Colic-Peisker and Deng, 2019). Their usual migration pathway is via the skill or investment visa streams.

When it comes to African migrants, they are from a vast continent of 54 different countries. These countries are not considered to have the economic and political clout that China has. Although the African continent is home to nearly one-third of the estimated 7,000 languages in the world (Wolff, 2021), the languages of power are non-African, rather they are languages of former colonial powers (e.g., English, French, Portuguese, and Arabic). Nevertheless, Africans are mostly bi/multilinguals, and they rely on oral tradition-based heritage languages as well as socially learned lingua franca for intergenerational communication and socialization. Multilingual repertoires are part of their everyday norms, and traditional African societies are known to have “their ways of educating their children by introducing them, playfully and through language (through tales, songs, riddles, proverbs, and language games), to culturally relevant concepts and value systems” (Wolff, 2016). In terms of language and formal education, the imposition of colonial languages and their subsequent position as formal and/or official language have led to literacy being taught in these foreign languages as the medium of instruction (Obanya, 1999; Ouane and Glanz, 2010).

The Chinese and African immigrants, similar to all other migrants to Australia, with their diverse languages and ethnic backgrounds, have brought vitality to a multilingual and multicultural Australian society. They form an important part of Australian demographic dynamics, especially in terms of identification with their home countries and Australia, language ideologies, educational needs, and orientations. In this study, we aim to deepen the knowledge about Chinese and African families' experiences with regard to emotionality and heritage language maintenance.

## 2. Theoretical framework

### 2.1. Family language policy, heritage language outcomes, and power relations

Family language policy (FLP) refers to “deliberate and observable” as well as “default and invisible” planning in relation to language choices, uses, and practices specifically within the home domain (King et al., 2008; Curdt-Christiansen, 2009, 2018). The tripartite model of FLP (Spolsky, 2012), which comprises language ideology, language practice, and language management, largely frames existing scholarship on heritage/minority language maintenance, parent–child interactions of immigrant families, and child bilingual development (Wang, 2017; Curdt-Christiansen, 2018; Shen and Jiang, 2023). Language practice refers to what families actually do with language, i.e., what choice they make from their linguistic repertoire; and language management is conceptualized as specific efforts or strategies they make to implement their language practice (King et al., 2008; Shen and Jiang, 2023). Underlying the two components is language ideology, the driving force of language policy regarding families' decisions and planning for the use of languages (Curdt-Christiansen, 2018; Shen and Jiang, 2023). Immigrant families, regardless of their ethnic and linguistic backgrounds, conventionally relate the rationale of heritage language maintenance to ideological beliefs of language as the symbol of identities, as the tie of families, and as the vehicle for economic empowerment (Borland, 2006; Et-Bozkurt and Yagmur, 2022; Romanowski, 2022). Taking the ethnic minorities in Australia as an example, most of the second- and third-generation Turkish parents in Melbourne related the responsibility of maintaining heritage Turkish to the survival of Turkish identity, preservation of Turkish culture, and communication with homeland relatives (Et-Bozkurt and Yagmur, 2022). Similarly, Polish–Australian fathers actively engaged with their children's Polish learning with the hope to safeguard their Polish identity, maintaining family ties in Poland and gaining bilingual competitiveness (Romanowski, 2022). What strategies the families adopt and what actions they take largely determine whether heritage languages can be maintained or developed in the younger generation. In the case study of three Chinese children and their families in Australia, the confidence and competence of Leo's (one subject child) heritage Chinese was associated with the high level of parental agency in language management, such as providing books in Chinese classic literature, reading and discussing the characters with the child, and watching television in Mandarin Chinese (Shen and Jiang, 2023). As a result, FLP provides the critical domain (Spolsky, 2012) or the cornerstone (Et-Bozkurt and Yagmur, 2022) of the success of intergenerational language transmission.

In fact, FLP, being a private family matter (Anthonissen and Stroud, 2022), is a socio-political reflection that gives priority to social utility, language prestige, educational empowerment, and socio-economic gains (Wang, 2017; Curdt-Christiansen, 2018; Curdt-Christiansen and La Morgia, 2018). The direction of language shift usually occurs from the minority language to the majority language or from the lower-status language to the more prestigious high-status language (Et-Bozkurt and Yagmur, 2022). Although the majority of Turkish–Australian parents believed that the Turkish language was important as it interwove Turkish identity and culture, they did not consider Turkish to be in a position to compete with English (Et-Bozkurt and Yagmur, 2022). For the best of children's economic future, they accentuated the significance of higher skills in English, viewing it as key to good education and social mobility in Australia (Et-Bozkurt and Yagmur, 2022). Such value-laden language ideologies are often well-represented from power-inflected language planning and decisions at the family level within the broader global spaces. For instance, middle-class families in China appropriated differentiated agencies in dealing with three languages: Fangyan, Mandarin, and English (Curdt-Christiansen and Wang, 2018). Parents often chose to let go of intergenerational transmission of Fangyan but placed great emphasis on their children's Mandarin and actively invested in their study of English. For parents (and children), Fangyan, though an important vehicle of parental emotionality, was linked to locality and impracticality, Mandarin to prestigious position and symbol of Chinese identity, and English to global mobility and international integration. In African countries, a hierarchy of languages exists in the form of a three-tiered “linguistic pyramid” whereby the languages at the apex [official languages, e.g., English, French, Portuguese, and Arabic (retained languages from colonization and other forms of contact)] are endowed with higher status than languages in the middle (Lingua franca, e.g., Kiswahili) and significantly greater prestige than the base languages (over a 1,000 distinct heritage languages; Obanya, 1999; Wolff, 2021). The languages at the apex are the ones linked to prestigious positions and “used for education, business, and government affairs,” so “mastery of these languages is closely related to educational attainment and occupational/social status” (Obanya, 1999, p. 14) and to international mobility and education. The remaining languages mainly function as important vehicles of affect and parental emotionality and hold grounds for the creation, perpetuation, and maintenance of traditional artifacts, arts, and history, which end up forming the bedrock of information for most scholarly studies (Obanya, 1999). These languages also form the basis of African parental influences and child socialization practices in the Australian migration context (Ndhlovu, 2014; Mugadza et al., 2019; Akosah-Twumasi et al., 2020) where they tend to be mostly invisible in public domains. Thus, the study of family language policy should recognize the relevance and influence of visible and less visible political, social, educational, and economic forces in a given society (Curdt-Christiansen, 2013).

### 2.2. Language emotionality, heritage language maintenance, and societal language mastery

The terms “emotionality,” “emotion,” and “feeling” can be used interchangeably, as shown in the psychology of language learning research on emotion (Sevinç and Mirvahedi, 2022). For this study, we draw on emotional descriptions in the ethnographies of Sevinç (2020) and Wang (2022). Therefore, we use language emotionality when referring to emotional nature or quality in relation to language acquisition and practices. For language emotions, we focus on specific types of feelings about languages such as happiness, excitement, or anger.

The abovementioned ideologies, either associated with family, identity, or power, are intimately involved with people's internal emotional worlds. They are affected by and prompt different types of emotions (e.g., affection, intimacy, satisfaction, anxiety, stress, and distance), regarding heritage language maintenance or shift. Situated in Australian contexts, the Polish father's constant engagement with their children's daily activities, through the use of the heritage Polish, fostered intimate communication and constructed mutually positive feelings (Romanowski, 2022). Similarly, the connection between the Australian-born Chinese children and their grandparent generation relied heavily on heritage language, which served as an expression of love and a bond of affection (Shen and Jiang, 2023). Conversely, the immigrant parents living in Sydney, despite their various ethnic origins, found that the language shift not only erected a kind of fence or barrier between their past and their present but also established emotional distance between them and their children (Tannenbaum, 2005). Parents, regardless of their differences in cultural backgrounds, generally felt depressed or rejected if their children did not speak the minority language that parents addressed them in and felt ashamed at their children's lack of heritage language proficiency (De Houwer, 2017; Sevinç and Dewaele, 2018; Sevinç, 2020). At the same time, children often felt stressed, unhappy, and even angry at being forced to learn the heritage language (Sevinç, 2020). The emotion-laden conflict in language preference and habitus can be a result of the intergenerational divergence of bilingual repertoires. The first-generation parents generally feel comfortable speaking to their children in their home or minority language as it is natural, spontaneous, and more connected to their inner world, while their children (e.g., 1.5 or 2nd generation) tend to use majority language more habitually or skillfully (e.g., Sevinç and Dewaele, 2018; Wang, 2020). Given the intimate link between emotional loading and stronger language, parents and children tend to be less well-attuned to each other's emotional world, leading to a discrepancy affecting the family dynamics (Pavlenko, 2004; Sevinç and Dewaele, 2018).

The emotional upheavals suggest a potential universal that parents of the minority language, regardless of their ethnic backgrounds, wish their language to be passed on to their children. At the same time, they want their children to do well in the societal language (De Houwer, 2017). In the exploration of mothers' global satisfaction regarding their bilingual rearing, although there were feelings of awkwardness linked to the assumed failure in transmitting the minority language to their children, there also was a high level of satisfaction largely based on the perceived progress of child bilingualism as a whole (Leist-Villis, 2004). When enforcing FLP, parents felt insecure about or even torn by how to balance the wish for their children's inheritance of the minority language and the desire for their children's mastery of the societal language (Sevinç, 2016, 2020; De Houwer, 2017). In immigrant and minority contexts, parental language planning and decisions, which often generated a full range of emotions, were situated in the battlefield of competing priorities of heritage and societal languages (Sevinç, 2020). In many cases, parental anxiety about their children's integration into mainstream society or their compromise on their children's language shift may cause parents to forego language maintenance goals (e.g., Tannenbaum and Yitzhaki, 2016; Wang, 2020). For example, Arab transnational families living in Israel tended to send their children to Hebrew-speaking schools, even with an awareness of the potential consequences of emotional prices from the compromise on Arabic language fluency, religious beliefs, and value systems (Tannenbaum and Yitzhaki, 2016). These educational decisions were primarily based on the parental valorization of Hebrew as a good investment into a more secure education and better assimilation. Therefore, FLP, which prioritized societal Hebrew over heritage Arabic, underscores the significance of power relations in shaping language ideologies, language planning, and decisions.

Thus, the central themes that emerged from the foregoing scholarship suggest that whatever pattern language maintenance takes, decisions usually rest on a rather strong emotional basis. To the best of our knowledge, emotion research in relation to the heritage language is gaining currency, but it is heavily shaped by quantitative frameworks (e.g., Xiao and Wong, 2014; Luo, 2015; Jee, 2016, 2020). The few lived experiences presented in qualitatively informed language-related emotionality are usually limited to a few ethnic groups, such as the Turkish families' language anxiety about the use of minority Turkish and majority Dutch in the Netherlands (Sevinç and Dewaele, 2018) and Mongolian women's emotional relief when translanguaging in Australia (Dovchin, 2021). Our study provides an ethnographic exploration of the emotional nuances of Chinese and African families in the context of Australia.

As mentioned earlier, language emotionality is frequently prompted by language ideologies, language behaviors, and perceived outcomes (e.g., Tannenbaum, 2005; De Houwer, 2017). Thus, the study examines the attitudes and practices that Chinese and African families have held and employed. This will give context to interpreting the resulting emotions in the enforcement of FLP. Due to the significance of FLP in the emotional and linguistic stability of transnational families and their children (Romanowski, 2022), this study broadens the scope by investigating the intricacies of language-related emotionality experienced by Chinese and African families in Australia. Our study also provides a comparative ethnography of language ideologies, language practices, and parental emotions, in relation to the heritage language maintenance of these two immigrant ethnic groups. In particular, it reveals how power relations play out in the similarities and differences of language practices and emotional experiences of these Chinese and African families when supporting bilingualism in relation to their heritage languages. The research addresses the following questions:

1. What language maintenance attitudes and practices can be observed in Chinese and African families?
2. What language emotions are emergent on the part of Chinese and African parents in the process of heritage language maintenance?
3. How does the study's Chinese and African parents' emotionality interact with their language ideologies and power dynamics?

## 3. Methodology

Our research is a comparative study of language emotionality emergent in two ethnographies of Chinese and African migrants living in Australia. In this study, we reuse, share, and analyze data pooled from the two ethnographies. The first ethnography documented Author-1's investigation of specific emotional discourse related to FLP and maintenance experiences (see Wang, 2022). This formed an extension of her PhD project which investigated Chinese heritage language maintenance trajectories in Australia (Wang, 2020). The second ethnography was drawn from Author-2 and Author-3's Hidden Oracies project. It was an extension of Author-2's PhD thesis (Williams Tetteh, 2015), which investigated African families' language maintenance and language use in their settlement trajectories, particularly the extent to which these hitherto invisible languages are used in Australia.

Our methodological approach of sharing and reusing data follows this emergent trend within the humanities and social sciences where qualitative data are being pooled, shared, and reanalyzed to paint a broader picture in ethnographic research about language and migration (see Piller et al., forthcoming). As such, while language emotions in interactions (e.g., when happy or angry and when satisfied or disappointed) were not key foci for both research projects we draw from, language-related emotionality did, in fact, loom large in both as we found in our field notes and through various discussions about our projects. Initial conversations we had as research colleagues showed some commonalities and differences in our datasets worth pursuing as a broader and comparative study. As mentioned in the introduction, some African parents would at times make references to Chinese families when comparing their families' linguistic and migration challenges. Thus, we pooled both datasets together for reuse, and we systematically analyzed the data, which brought forth the parents' overt and implicit emotions in relation to heritage language maintenance as migrants in Australia. These formed the basis of numerous follow-up discussions we had about the interpretation of our shared data and the research findings we present in this study.

### 3.1. Participants

The participants in the study are from Chinese and African families recruited through referrals from the community or research colleagues who know the families and the criteria set out in our recruitment advertisements and by word of mouth. The families we engaged with for the study are well-educated middle-class Chinese and African parents who immigrated to Australia in recent decades. There were 25 migrant parent participants in the study (see Table 1). In total, 13 parents (three fathers and 10 mothers) migrated from China. Of the remaining, 12 (four fathers, five mothers, an uncle, and an aunt) migrated from different parts of sub-Saharan Africa, namely Zimbabwe, Ghana, South Sudan, and Rwanda, and one of the fathers was Australian-born. In total, 21 parents held bachelor's degrees or above, three (one Chinese and two African) held vocational diplomas, and one African parent had up to year 10 schooling equivalent. Notably, 22 of them migrated to Australia between 2000 and 2017, and only three (one Chinese and two African) migrated in the 1990's. Before migration, all of them worked in professional roles in academia, government, NGO, finance, IT, or health. These families had 24 school-aged children in total, ranging in age from 8 to 21 years. They attended either primary school or high school at the time of the interview with the exception of two who were university students. All the names used in the research are pseudonyms. Chinese participants' pseudonyms include both the family and given names, and African counterparts' pseudonyms only include given names.

**Table 1:** Summary of parents' migration backgrounds and languages.

**Country of origin**	**Migration period**	**Parents in study**	**Children in study**	**Languages spoken**
China	2007–2014	Three fathers; 10 mothers	Seven sons; seven daughters	Mandarin, English, Cantonese, Shanghainese, Sichuanese, Hakka, and Hokkien
Zimbabwe	2000–2017	Two fathers; two mothers	Three daughters	Shona, Ndebele, and English
South Sudan	2000–2008	Father; mother; aunt	Two sons	Arabic, Madi, Luganda, Kuku, Swahili, and English
Ghana	1990–1994	Father; mother	Daughter; son	Ewe, Ga, Akan, Pidgin English, French, Spanish, and English
Rwanda	2006	Father^*^; mother, uncle	Daughter	French, Swahili, Kinyarwanda, Kirundi, Auslan, and English

^*^Father is Australian born of Anglo origin.

### 3.2. Data collection

As mentioned above, data for this study are pooled from two ethnographies of African migrants and Chinese migrants in Australia. Ethnographic data gathered for both studies include transcriptions of open-ended semi-structured interviews with parents and children, field notes from informal conversations and observations, photographic evidence of children's literacy resources and practices, and language portrait (LP) descriptions. The LP method derives from a multimodal research tool, where both the visual and verbal modes play a role in constructing the participants' identity, language ideology, and attitudes as well as their lived language experiences and emotional states (Busch, 2012, 2016; Obojska and Purkarthofer, 2018). It goes beyond the languages used to express cognitive, emotional, and lived experiences (Busch, 2012, 2016; Wolff, 2016). For the present study, data from children and LPs were not included in the analysis.

The data for Chinese families were collected between 2017 and 2019 and for African families in 2019–2020. All interviews with Chinese parents except one (with Ge Chang) were conducted in Mandarin Chinese. Ge Chang preferred to be interviewed in English. Interviews with African parents were in English. All interviews were transcribed verbatim. Field notes from informal conversations and observations were noted down in Chinese and English, respectively. The non-English data selected for analysis were translated into English.

### 3.3. Data analysis

Data analysis followed previous ethnography models from previous studies (Tannenbaum, 2005; Tannenbaum and Yitzhaki, 2016; Sevinç and Backus, 2019). We have used inductive thematic analysis as the major analytical method to establish patterns of language use and participants' interpretation of their repertoires in relation to their settlement in Sydney and Australian society. The analysis in this study mainly addresses the themed areas based on the centrality of the abovementioned research questions: What feelings do parents express about languages and how do these reflect their emotive states? The transcript and field note data that conveyed parents' emotionality were initially coded into concrete themes such as oral language use, literacy language practice, children's favorable attitudes, children's resistance, proficiency outcomes, language as investment, parental happiness, and parental struggles—in NVivo. The emotional expressions were visible through the parents' use of sentimental words (e.g., regret, annoyed, upset, enjoy, proud, and amazing) or through the emotional behavior they displayed (e.g., speaking with tears in their eyes or with laughers and beaming with smiles) when they recounted their language maintenance journey. Since it was not always possible to thematize data in a clear-cut way, some data were coded with more than one theme. These themes were then allocated to the main categories including heritage language practices, parents' language ideologies, and parents' emotional experiences, as reflected from the titles of the following data analysis sections. In addition, data from collected evidence of FLP and maintenance results, such as photographic images provided by Chinese families, were placed into categorized files and titled “Chinese literature books,” “Chinese writing samples,” “certificates and awards,” “school reports,” and so on. The purpose of the thematization and categorization was to conceptualize immigration narratives, language use patterns, language attitudes, and negative/positive feelings and then to identify associations between heritage language issues and familial emotions of parents in a migration and minority status.

## 4. Findings: language maintenance and parental emotionality in the discourse of Chinese and African families

### 4.1. Language maintenance attitudes and practices of Chinese and African families

In the exploration of heritage language maintenance experiences of the subject families, there emerged similarities as well as noticeable differences between Chinese and African families in terms of their attitudes to and practices of language maintenance. Both Chinese and African families aspired to pass on their heritage languages to the next generation and the parents typically expressed their desires as follows:

She [Cai Xi] should speak Chinese. Or it would be so weird that a Chinese person can't speak Chinese. (Cai Wei)

I think it's always been dreams[sic] like to keep in my culture, my language. (Jeanette)

Across the data, parents, regardless of their ethnicity, clearly stated their affection for their heritage languages. Both Chinese and African families had made efforts in maintaining their children's heritage languages, primarily the oral skills, and parents talked about how they pushed their children to speak their languages in daily communication:

I always say, “no English at home.” They [Ge Si and Ge Bai] are not allowed to speak English to each other. When they speak English, I say “STOP.” (Ge Chang)

I tried to speak Shona to [Child name] every Friday when I don't go to work […] Just to make sure that this child keeps speaking Shona but I can FEEL it[sic] that I'm fighting against […] all odds. (Bandi)

As shown above, parents usually needed to fight against a child's habitual use of English when they endeavored to maintain the child's heritage language oracy. The parental struggle in language maintenance reveals how difficult it is to keep minority languages in a monolingual mindset society (Clyne, 2008; Piller, 2016), even at a basic communication level, let alone the aspect of reading and writing.

**Figure 1:**
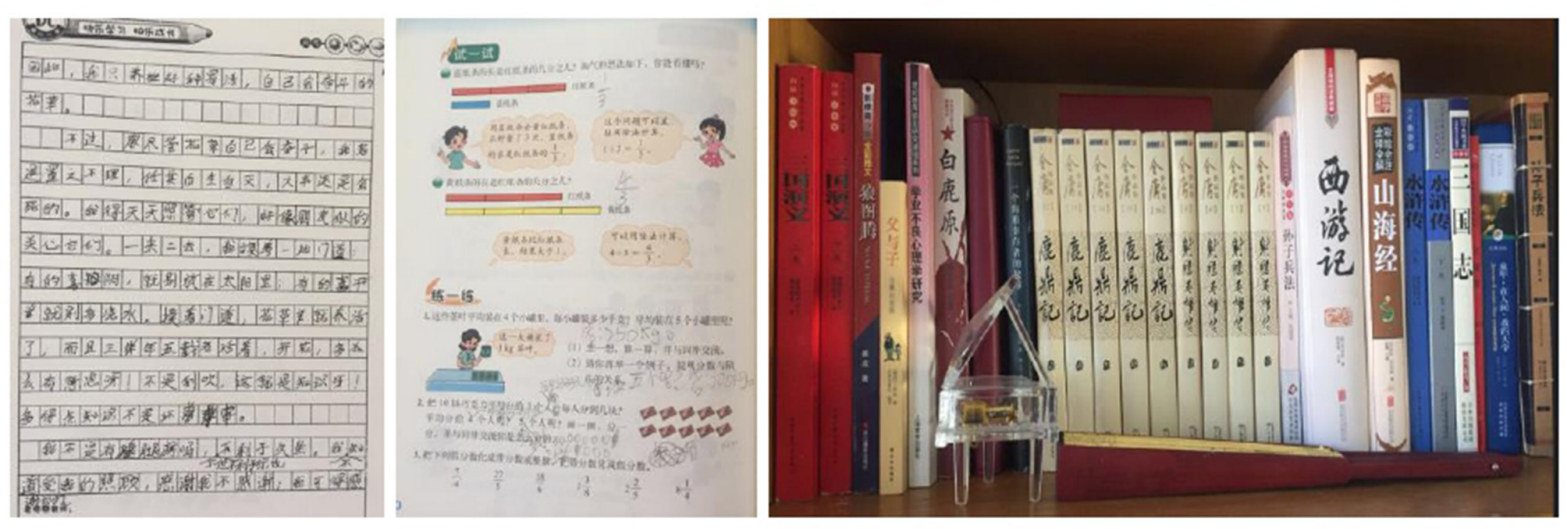
A sample from Ji Ming's copied texts **(left)**, math mark **(middle)**, and reading collection **(right)**.

However, in terms of heritage language literacy maintenance, there emerged a striking difference between Chinese and African families in their investment in their children's reading and writing. In this study, Chinese parents' heavy investment in their children's Chinese literacy forms a contrast with their African counterparts' more lax attitudes to the literacy development of their languages. The Chinese immigrant families widely involved their children into various literacy practices, which included reading Chinese literature, writing Chinese characters and essays, practicing calligraphy, and doing Chinese math (also see Wang, 2020). In the process of literacy involvement, these parents used Chinese textbooks, exercise books, and literature materials from China as important resources for a home tutoring or for assisting with community school assignments (see Figure 1), as referred to by Ji Ran—Ji Ming's mother:

Every time my friends went back to China, I asked them to bring us Chinese books, like the[sic] textbooks, math books, and lots of novels. My son [Ji Ming] is requested to copy one Chinese text each day and to do math exercise in[sic] school holidays. He is also encouraged to read more Chinese novels—whatever he likes. He read quite a few sets of Gongfu [功夫, Chinese martial arts] novels written by Jinyong [金庸 —a well-known Gongfu novelist in Hongkong]. He also read all the[sic] Four Great Classic Novels I recommended. That's why his Chinese still improves[sic] in Australia, especially in the aspect of comprehension and general knowledge. (Ji Ran)

Ji Ming's parents' effort of obtaining various social (friend's help) and linguistic (e.g., Chinese and math textbooks and classic literature) resources is not exceptional among Chinese families. Across the data, Chinese textbooks (including math) and/or different kinds of reading materials, usually brought from China or bought online, were used to different degrees by Chinese families. For example, the families (e.g., Ge Bai's and Shi Diwen's) who used math Chinese books often emphasized the dual function of Chinese learning and math advancement, which was often described as “一举两得 ‘kill two birds with one stone'.” Most Chinese parents evidently considered their children's literacy proficiency as the crucial marker of the success or failure of their family language policy or language maintenance outcome. When Xu Li's mother, Xu Dai, admired other children's ability in reading sophisticated books, she concluded with a sense of loss that her language tutoring was unsuccessful because “Xu Li's vocabulary remained stuck on grade-one level.” In the Chinese diaspora, parents' utilization of multiple resources, their consideration of language planning, and their emotions from children's language proficiencies, as manifested in the above families, reflected a typical aspiration for Chinese language proficiency, especially literacy competency (Li, 2006, 2007; Wang, 2020).

In contrast with the recorded rich resources employed and the tight schedule made by Chinese families in literacy practices, African families scarcely reported their aspiration for or efforts of developing their children's literacy competence. In the data, Ruth is the only parent who reported that she had attempted to source reading materials in Madi, her heritage language, for her children, Isaiah and David. Even so, her focus on language development with the children was by speaking in Madi at home and making them participate in weekend community activities to maintain the spoken language and ethnic culture. In effect, the divergent attitudes in relation to literacy practices between Chinese and African families are deeply grounded in the linguistic and historical background of their communities as well as the entrenched linguistic hierarchies in the transnational market (see details in the Conclusion section).

In sum, both Chinese and African parents attached importance to maintaining their children's heritage languages and attempted to implement the rule of speaking-only-Chinese/African language(s) at home, but with reference to literacy practices that these parents employed in Australia, Chinese parents, in comparison with their African counterparts, demonstrated greater concerns for their children's reading and writing and made significantly more efforts into developing their children's literacy skills in the heritage language. This noticeable difference should be situated in both the micro-discourse of Chinese and African families as well as the macro-discourse of Chinese and African communities as well as broader societal contexts.

### 4.2. Parental emotions of heritage language maintenance

In the exploration of the language maintenance experiences of all these Chinese and African families, a full range of parental emotions emerged. This section illustrates the emotional complexities, underlying factors, and arising consequences, by exploring first negative emotions typically known as anger, disappointment, and shame, which then shifts to positive sentiments such as joy, accomplishment, and pride.

As mentioned in the previous section, maintaining Chinese and African heritage languages is a desired family action, but the maintenance endeavors are often accompanied by stress-triggering experiences, such as children's unfavorable language attitudes and perceived difficulties in achieving optimal results, which often generate unpleasant feelings and impinge negatively on family cohesion. Parents, such as Shi Fan and Bandi, experienced such sense of anger and frustration:

My son [Shi Diwen] doesn't want to work hard at[sic] Chinese language. His dad at times got[sic] annoyed and said[sic] loudly: “Oh, your handwriting looks so ugly, you must WRITE, WRITE, and WRITE!” (Shi Fan)

You can hear Sandile is very fluent in Shona. But at that age, she also feels that she's got to be like others so she PRETENDS that she CAN'T speak Shona when I spoke Shona to her [frowned]. (Bandi)

The emergent conflict between parental expectations (e.g., doing regular Chinese homework or speaking Shona at home) and children's language behavior (e.g., reluctance to write or speak) becomes a frequent cause of unpleasant emotions or intergenerational clashes. When parents confront undesirable results of children's heritage language performance, they may blame themselves or may experience being blamed for their inadequate parenting. Ruth provided an example of how her sister-in-law's twin children felt upset at being unable to fulfill an undergraduate classroom task in relation to heritage language use and how they felt unhappy about not being brought up in their heritage language. Ruth's concluding comments on the story were “The mother and the father doing big mistake. Now, they regret it.”

When expressing a sense of disappointment, parents also blamed the school system for not providing (proper) heritage language education. Perceiving that the potential loss of the heritage language was due to parents' heavy work commitments and limited time availability, Mandla blamed the school for failing to take the leading responsibility of teaching African languages in formal school settings:

We don't have that privilege [economically] and [clears throat] so as much as we want that's why if it was taught in school it would be an advantage to us. We actually need that help to augment our efforts to make it happen. Because we are economic refugees. So our time with our kids as they grow up to actually nurture them language wise is very very[sic] limited compared to other people. (Mandla)

What we have seen is how parental anxiety about the unfeasibility of enacting language maintenance practices is caused by the perceived disadvantage of migration status (e.g., heavy workload and economic difficulties), changes in family dynamics, and a challenge in parental authority when migrant children assume the role of “language brokers” (Renzaho et al., 2017, p. 14), as well as the widely acknowledged lack of minority language support from the institutional level (Lo Bianco, 2009; Piller, 2016). For parents, such as Mandla, the financial burden has barely left them time for nurturing their children's heritage languages, and schools' neglect of African heritage languages further sped up children's heritage language loss. In fact, Australia has laid out various policies to support community language education, but resources are heavily focused on the languages ascribed with more economic status, such as Chinese, Japanese, Korean, and Indonesian (see Lo Bianco, 2009). This means that for African languages and many other minority languages with less economic capital, institutional and societal support is actually limited. Therefore, for families from those language backgrounds that are typical in south–north migrant realities, the intergenerational transmission of their heritage languages becomes greatly challenged (Kamwangamalu, 2013).

However, the maintenance of Chinese and African heritage languages in a migration context may not always lead to stress and anxiety. A sense of enjoyment, fulfillment, and pride, in relation to language maintenance practices and achievements, has also been identified in parental discourses. Across the data, parental positive feelings were closely related to the progress of children's fluency and literacy, as well as the endorsement of parental efforts within and beyond family domains. Mandla, Sandile's father, recounted a pleasant surprise at Sandile's improvement in speaking Ndebele after she was sent to stay with her grandparents:

The vocabulary that she'll be speaking[sic] you'll be like wow. That's when I realized, my parents had a huge influence on her language. And she would speak words that her mother being half Ndebele half Shona sometimes she wouldn't understand. (Mandla)

In the migration context, where generational communication is often disrupted due to children's loss of heritage languages or shift to dominant languages (Fillmore, 1991), the intergenerational transmission of heritage languages which should have strengthened (grand)parent–child ties and family cohesion is often missing. Parents generally feel close to their children when both parties speak the same language and/or feel respected when children endorse parental language policy and show favorable attitudes to their heritage languages. Ruth revealed such enjoyment with a tone of contentment, “All the time I speak in Madi with my children. Yeah. And they like it.”

Children's achievements in their heritage languages, if acknowledged by their transnational families, ethnic communities, or within institutional settings, do generate a greater sense of parental pride of accomplishment. Ruth's sense of pride seemed ignited when the whole family cheered for her sons' using fluent Madi to talk with family members on the phone:

Uncle and grandma,[sic] they are VERY happy. Yeah. they say I'm very proud of you. You teach your kids with[sic] our language. My uncle in Botswana, when he ring and talk[sic] to my kids, to,[sic] in my language, and he's so happy. He say[sic], [name] I'm very proud of you because you'll never forget to teach your kids with the Madie. Thank you for yours […] Oh, my God. You can't even believe, it is so nice. (Ruth)

**Figure 2:**
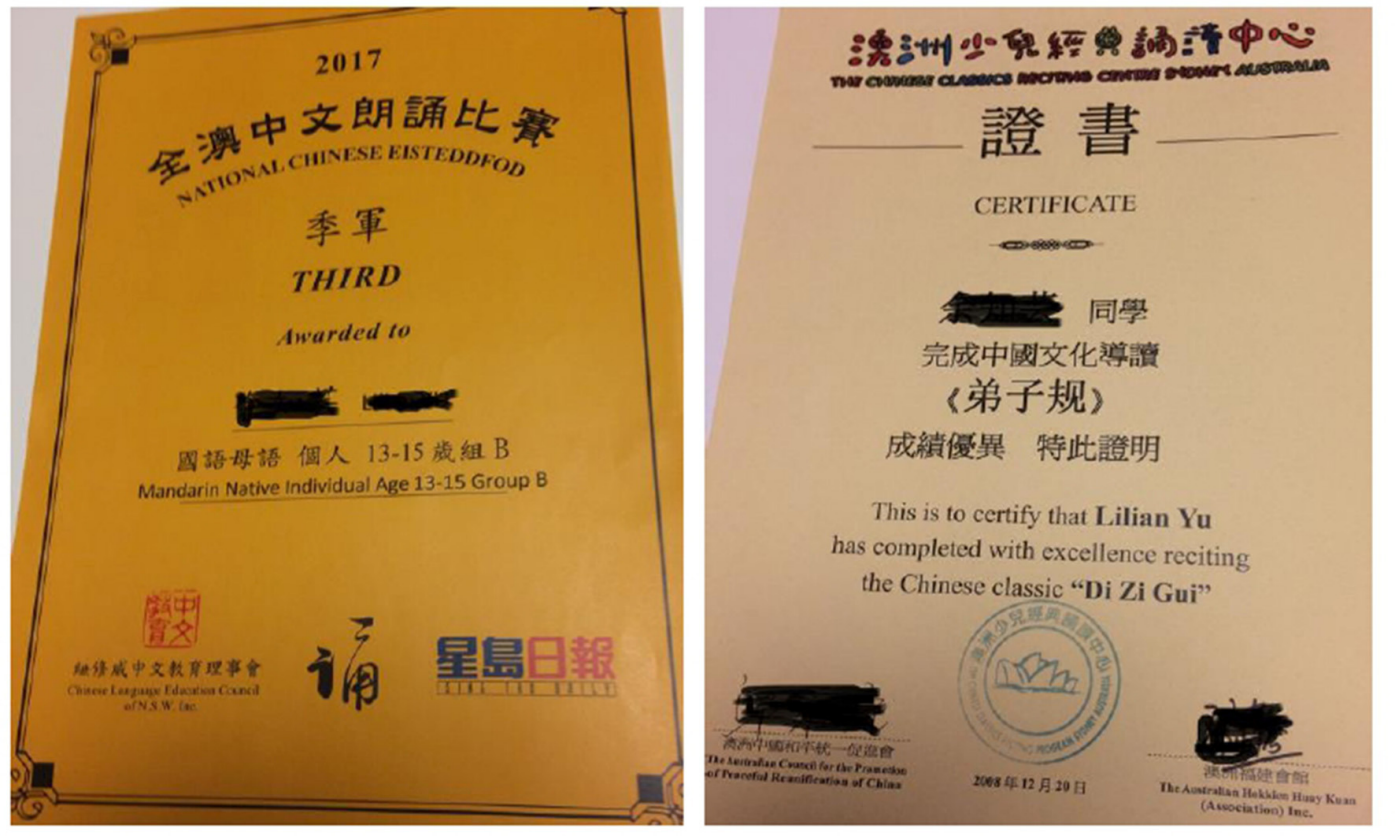
A sample from Li Long's awarded certificates in Chinese language activities and competitions.

What has been conveyed, from the frequent use of interjections such as “very happy,” “very proud,” “so nice,” and “my God,” is not only parental feelings of joy, gratification, excitement, and pride but also the important role of language in connecting family members and strengthening family ties. More importantly, the wider endorsement of the heritage language from the social and institutional level significantly enhances parental motivation for achieving higher-level proficiency and begets further success. Li Ni, for example, when expressing her satisfaction with the result of her family language policy, proudly showed evidence of this in a couple of certificates awarded to her daughter—Li Long, in various Chinese language competitions (Figure 2). She related her gratitude specifically to the support from Li Long's Chinese community schools and other language organizations, as she said, “A word of praise from teacher or a small reward from school is more than a thousand words from parents.” Against the widely assumed fact of language loss among three generations (Alba et al., 2002), the potential for a benign circle to operate confirms the feasibility of intergenerational transmission of heritage languages and underscores the significance of concerted efforts from institutions, communities, and families.

It should be noted, as mentioned earlier, that due to different historical backgrounds and linguistic situations, Chinese parents, compared with their African counterparts, demonstrated greater aspirations for and efforts into maintaining their children's literacy competency which can explain the reason why parental emotionality related to Chinese language maintenance is largely associated, in addition to oral-based skills, with parental expectations for children's literacy competency.

In sum, the experience of heritage language maintenance is fraught with emotions, negative (e.g., anger, frustration, regret, and disappointment) and positive (e.g., enjoyment, excitement, fulfillment, and pride). Language emotionality seems to interplay with parental expectations, maintenance results, and children's language performances. In fact, what underly parental emotionality of the heritage language is their language ideologies, particularly, ideologies of power relations. We will demonstrate this in the following section.

### 4.3. Parental emotionality and language ideology in relation to power

Across the data, parents' emotional realities and maintenance practices, though related to the consideration of ethnic identity and familial bond (Wang, 2022), are heavily influenced by the concern of economic returns from language investment. This capitalist appraisal of the heritage language reflects the multifaceted layers of inter-power relations between English and minority languages, Chinese and African languages, and intra-power relations within various Chinese or African languages.

Chinese parents, almost in common, relate their motivations for Chinese language maintenance, for the purpose of reaping the economic, occupational, and educational benefits of Chinese in transnational diasporas. These parents, such as Xia Tian's father—Xia Ming, specified the usefulness of learning Chinese with a focus on the socio-economic prospects of China in the global world:

China plays a more and more important role in international[sic] world, whether in economic or political position. When children grow up, they definitely have chances to work with Chinese, whether in Australia or in China. Chinese is useful and I have confidence. (Xia Ming)

The above quote shows how the political and economic status of a home country (China) empowers its social agents (Chinese immigrants) in migration contexts (Australia) to take action of maintaining their language. In fact, Chinese parents across the data demonstrated a detectable sense of pride in the emergent prominence of their heritage language (Mandarin) as well as a sense of urgency to harness the economic edge in the growing Chinese market. However, no matter how desirous Chinese parents were for their children's competence in Chinese, particularly a functional or high level of literacy, they were often caught in a dilemma when struggling to balance their children's learning of heritage Chinese and school subjects—usually taught in English. As a result, parents generally compromised the value of Chinese for the purpose of achieving academic excellence in schools as they feared that the time spent on the Chinese language would jeopardize their children's performance in high-stake assessments [e.g., tests for opportunity classes (classes in years 5 and 6 which are designed for gifted and talented students), tests for selective high schools (high schools for academically gifted students), and HSC (the higher school certificate)^1^ examination]. Although Xu Li's mother, Xu Dai, admitted that it was “a pity” to discontinue, for quite a few times, Xu Li's Chinese learning during preparation toward critical examinations, she firmly stated that the suspension was “the only choice” they could make because Xu Li needed to “make concentrated efforts” to be well-prepared in year 4 for the test of opportunity class, in year 5 for the test of selective high school, and from year 10 for HSC examination. Such inconsistency or conflict in maintenance practices, though implemented with more or less feelings of regret (as shown by Xu Dai), was generally described by parents as “a wise decision for the best of child's education.” Parental attitudes, decisions, and practices in relation to their heritage languages seemed well-constructed on the power structure between majority languages and minority languages, in which English—the majority language in Australia and the lingua franca in the world—enjoys unique prestige above all other languages in and beyond educational discourses; that is, while Chinese is much valued for its rising currency, English is unanimously recognized as the language carrying the maximum weight in child's immediate education and in the more distant future. The stereotypical view of “superior” over “inferior” languages is explicitly or implicitly represented in language attitudes in the broader African diaspora and influenced the subject African families. The view of English as superior seems deeply ingrained in the social and educational discourses of the African population. Mandla pointed out the pervasive admiration of English back in his home country as follows, “English is admired, everything English feels prestigious. Everything. English is better.” Due to the widespread “English fever” (e.g., see Cho, 2021) observation in the context of South Korea, English is prioritized as the desired means of children's education, as Mandla continued:

Everybody, who has a little bit of money back home sends their child to a group A school [top rating private educational institutions]. There are even Schools[sic] now back home that don't even consider Shona as a subject as a pass. If you don't have a Shona at your O level, it doesn't matter, if you fail Shona at your grade seven, it doesn't matter. You know, so that culture is ingrained in many Zimbabweans. (Mandla)

With the African families living in Australia, parents were divided into those who desired to keep their African languages and those who seemed to make flexible language policies forgoing their heritage languages. Parents admitted that they “never put them under pressure to learn Ndebele” (Lisa) or “If they can't speak you know Ndebele, then let them speak English. I'm okay with that” (Mandla). In terms of the causes to the lax attitude of maintenance policy, parents' own English/French medium education background and the limited social-economic gains from their heritage languages, especially in a society with English as the dominant language, posed as significant factors. Situated in a market with entrenched linguistic hierarchies, African parents, in general, did not hold an optimistic view about the prospect of their own heritage languages. Mandla, for example, felt sad about the unfavorable situation of maintaining the heritage language—Shona within the second generation:

If the mentality [admiring English and ignoring Shona] of the Zimbabwean here in Australia doesn't change, [sic]The next generation won't be speaking any Shona […]. The future of Shona in Australia is very dark. (Mandla)

In fact, African parents' pessimistic sentiments about the prospect of an African ethnic language form a contrast with Chinese parents' positive expectations of the Chinese heritage language in a migration context. The contrastive sentiment also reflects the hierarchical relationship between the Chinese language (more precisely, Mandarin Chinese) and African ethnic languages in the profit discourse where the desired former carries heavier currency than the lesser-desired latter. The instrumental hierarchies largely grounded peoples' attitudes to and practices of these languages in both parental and public discourses, as pointed out by Mandla:

You see, even Indians or Chinese people whatever Asians. Their young kids, you'll see them communicating in their vernacular languages. But if a Zimbabwean mom is speaking to a Zimbabwean kid, you'll think they're all Australians behind you if you don't look back. […] And when you turn back, they're just as black as you are.[sic] (Mandla)

In fact, influences on parental ideologies and emotionality not only arise from the globally entrenched power structure of English, Chinese, and African languages but also from the regionally based competition within minority Chinese languages and within minority African languages.

It should be noted, and as mentioned earlier, that the term “the Chinese language” is tacitly approved by all the Chinese participants as “Mandarin Chinese” rather than any other Chinese varieties. In the fieldwork, there emerged a clear consensus that, from the perspectives of many Chinese, Mandarin is considered a language while other Chinese varieties are considered regional dialects which may retain some economic or symbolic value but are not considered at par with Mandarin. The stereotypical conception of superior Mandarin vs. inferior others also explains why the Chinese parents spontaneously equated the maintenance of the so-called heritage language with that of Mandarin Chinese, regardless of their actual language backgrounds. This habitual use of terminology (e.g., which is regarded as the Chinese heritage language) reveals the hierarchical order between Mandarin—the prestigious national language, and other varieties—usually indexed to locality and lower status (see Wang, 2020). Based on the instrumental appraisal, Chinese parents' heavy investment into the heritage language was predominantly focused on Mandarin Chinese rather than their own heritage others if non-Mandarin. In the data, only two parents, Jie Yu (Cantonese) and Li Ni (Shanghainese), maintained the oracy of their heritage Cantonese and Shanghainese on a regular basis, while most other non-Mandarin heritage parents had foregone their mother tongues such as Hokkien (Xia Tian's family), Hakka (Mo Jie's family), Shanghainese (Cai Xi's family), and Sichuannese (Yang Mei's family). Parents' lax attitudes to their own spoken languages, in contrast with their devotion to Mandarin, further entrench the power gap between national Mandarin and regional others.

In fact, the aforementioned emotion, either joy or sadness, is in general related to the maintenance outcomes of the privileged Mandarin other than the inferiorized mother tongues. Apart from the pervasive favor of Mandarin, parents at times showed delicate (dis)favor to some specific regional “others,” which seems to reveal a delicate stratification between Chinese varieties other than Mandarin, as indicated by Li Long's mother:

We Shanghai people, more or less, have a sense of pride in being Shanghainese. So, I still want to[sic] my daughter to keep our language. But most of my Shanghainese friends have given up speaking Shanghainese with their children because they think Shanghainese is not that useful and Mandarin is the most important. (Li Ni)

Parents' (e.g., Li Ni) nuanced overtone of some regional Chinese (e.g., Shanghainese) and an undertone of others (e.g., non-Shanghainese other than Mandarin) not only define the distinction of Mandarin but also reveal an implicit power layer, which seems to put some sets of “regional varieties,” such as Shanghainese and Cantonese, ahead of similar others (also see Wang, 2020). The embodied language (dis)favor, largely power-oriented, further exposes the intricacies of hierarchical orders existing in Chinese languages, which put Mandarin at the unique top layer, then followed by Shanghainese/Cantonese due to their economic importance or symbolic value in China, and more others at the bottom level.

The nuanced layers of superiority vs. inferiority in minority African languages were observed from African subjects' language attitudes and emotional responses. For example, the Ewe parents, Phoebe and Efo, revealed their irritability at intra-community linguistic hierarchies that persistently positioned their heritage language as inferior within the Ghanaian community, what Efo described as being spoken to “as if who you are doesn't matter.” Both attributed this positioning to politics in their home country which continue to shape their interactions with the majority Twi or Akan speakers even in a migration context where both languages are constructed as minority languages. Experiences that they narrated included interruptions by onlookers at Ghanaian community gatherings where the majority of Twi-speaking community members expected Twi to be spoken. Phoebe told of how on one occasion an Akan woman butted into a private Ewe conversation yelling and demanding that they speak Twi, “HEY HEY HEY NO EWE NO EWE, SPEAK TWI.” Efo also recalled “several instances” at church where private Ewe conversations with his wife, Phoebe, were met with admonitions to speak in Twi, “Hey don't. Speak in Twi.” Efo explained further that because people knew them to be bilingual in Ewe and Twi, “We can understand their language, but they cannot understand ours,” and some of the Twi speakers felt suspicious when they chose to use Ewe and not Twi. This minority positioning within a minority language community is seen as demeaning and threatening to the upkeep of the Ewe language and their speakers' identity/dignity. This negative positioning concerned the parents so much that they expressed relief and praised the study for looking into shedding some light on such power-led linguistic issues faced by minority language speakers.

As illustrated in this section, parents' language ideologies, maintenance practices, and emotional responses are deeply grounded in the power relationships of languages both in global and regional discourses. The power structure revealed in the research not only entrenches the unique prestige of English and features the rising currency of Mandarin Chinese in the global world but also reveals a delicate stratification of minorities within minorities, either in terms of Chinese varieties or African languages.

## 5. Discussion and conclusion

This study documented language emotionality experienced by Chinese and African immigrant parents in their practices of maintaining their children's heritage languages. It explored how these parents' different emotions interplayed with their language ideologies in relation to power dynamics. Parental emotionality of heritage language maintenance manifested by these Chinese and African families accentuates three characteristics: shared aspiration for language maintenance and divided action in literacy practices, complexities of emotional experiences, and significance of power-inflected ideology in parental emotionality.

First, the shared aspiration for Chinese/African language fluency between Chinese and African families echoes with heritage language desires across ethnic and minority groups in the context of Australia and beyond (see Et-Bozkurt and Yagmur, 2022; Romanowski, 2022). The comparative investigation of the attitudinal divide in the aspect of heritage language literacy offers additional dimensions to FLP from linguistic, educational, historical, and political perspectives. In the research, Chinese parents, compared with their African counterparts, have displayed a stronger drive toward developing their children's reading and writing skills and have made heavier investments into their children's literacy development in the heritage language. This distinct divide in literacy desires and practices of their heritage languages is deeply grounded within the educational and historical backgrounds of Chinese and African diasporas. It reflects the hierarchical relations of languages in contexts before and after migration and closely associates with the differences in linguistic features between the Chinese language (Mandarin Chinese as referred to) and African languages. The Chinese parents received most of their education in China where Mandarin was predominantly used as the medium of instruction. The parents spoke Mandarin, along with some regional dialects if they had any, either in institutions or in private domains. However, the African parents, due to their home countries' colonial history, received their education in the medium of a European language, i.e., English, or French before their migration. The African languages were mainly learned through subject learning in school, Bible reading at the church, or daily communication, as some African participants (e.g., Phoebe) mentioned. For them, literacy gained in formal education is mainly tied to non-African languages (e.g., English and French), and heritage language maintenance tends to be oral-based and is usually tied to informal learning. Thus, the linguistic status in the educational systems of China and African countries underscores, respectively, the significance of Mandarin and European languages such as English and French. For African families, their previously held language habitus which prioritizes English seems further entrenched in Australia where literacy remains legitimately linked to English, and the use of heritage languages is largely confined to private domains. Where the written form is concerned, unlike Mandarin Chinese, which is standardized in simplified Chinese, many African languages do not have identifiable scripts to which their cultures and identities would have been tied. This constitutes another reason that their cultures, traditions, and values were handed down through oral communication and interaction, such as singing songs, telling tales, and remembering proverbs.

Second, the varieties and complexities of emotion types of the Chinese and African parents in this study have enriched the studies of language emotionality by complementing inquiries usually dominated by negative feelings, such as language anxiety experienced by Turkish families in the Netherlands (Sevinç, 2020) and by Korean families in Australia (Jee, 2020). This research brings forth a whole range of negative (e.g., frustration, disappointment, and shame) and positive (e.g., joy, fulfillment, and pride) emotions in FLP, as well as underlying reasons for such emotional dynamics. Parents' unpleasant feelings are mostly triggered by their children's resistance, undesirable outcomes, and perceived lack of societal support, while parental enjoyment and pride are attributed to their children's endorsement of FLP, their achievements of and progress in heritage language fluency, and/or literacy. The language-related emotionality, which looms large in migration contexts, indicates that heritage language transmission is significant to the psycho-social wellbeing of immigrant parents and their family cohesion (also see Wang, 2022). The difficulty of heritage language maintenance reveals the lack of institutional and societal support for many minority languages, especially those with limited instrumental capital, such as the African languages recorded in this study, while the positive feelings about the maintenance result suggest the potential for heritage language maintenance at the family level. The contrasting emotional experiences underscore the significance of combined efforts for heritage language maintenance from families, communities, and institutions.

Third, the value-laden ideology represented by parental emotions confirms the significance of power relations in the formation and implementation of FLP across ethnic diasporas (see Curdt-Christiansen and Wang, 2018; Et-Bozkurt and Yagmur, 2022). In a previous language research study, the documented power structure falls into the distinction between lesser status and more prestigious languages, typically between the majority language and the minority language, or between the official/“national” language (e.g., Mandarin) and the dialectal language (e.g., non-Mandarin). This research adds a new dimension by revealing the delicate stratification within lesser-role minority languages/varieties used in the Chinese/African diaspora. It is also the first study exploring the intersection of Chinese and African families in the same migration context (Australia) in relation to their languages and emotions. In the research, the multifaceted layers, which are based on linguistic utility, are reflected from the distinct hierarchy between majority English and minority Chinese/African languages, from a materialistic comparison between more profitable Mandarin Chinese and lesser “useful” African languages, and from the hidden tiers within Chinese/African languages/varieties. This practical ideology has significantly shaped the families' language decisions and practices. Both Chinese and African families prioritized “prestigious” English over their heritage languages through all stages of their children's education in Australia, though they must bear emotional costs arising from a child's language and culture loss. In addition to the linguistic and historical factors, the divergent aspiration for literacy transmission between Chinese and African parents can be a result of their practical appraisal of Chinese and African heritage languages. The rising currency of Mandarin Chinese strengthens Chinese parents' desires for literacy transmission, while the perceived “dark future” (as voiced by Mandla) of African languages (e.g., Shona) lowered parents' expectations for their children's heritage language proficiency. In addition, as the “national” Mandarin enjoys superior status over all other “regional” dialects in the Chinese language market, Chinese parents are willing to acknowledge Mandarin as the legitimate heritage that they should maintain rather than their own heritage varieties that are not Mandarin. It is largely the success or failure of Mandarin Chinese maintenance that generates a parental sense of fulfillment and pride or anxiety and shame. Even in terms of various “regional” Chinese, parents tend to elevate certain Chinese varieties (e.g., Cantonese and Shanghainese), which carry more materialistic or iconic weight and generate greater pride than other “regional” dialects (e.g., Hokkie and Hakka). In effect, whether or not to invest in Chinese heritage languages and which is the proper heritage language to invest in largely depends on the perceived economic returns in the market of Chinese languages. With their African counterparts, the intricacies of power structure embedded in African heritage languages in their home countries and in the diaspora deeply influence their choice of language maintenance and their emotional fluctuations toward their languages.

Therefore, themes emerging from the research suggest that the pattern of language maintenance and decisions in this regard are more than mere technical linguistic planning but could generate strong emotional reactions and reveal power hierarchies. Hence, the impact of language emotionality is essential for the psycho-social wellbeing of migrant families and has implications for policymakers and heritage language research.

## Data availability statement

The raw data supporting the conclusions of this article will be made available by the authors, without undue reservation.

## Ethics statement

The studies involving human participants were reviewed and approved by Macquarie University Human Research Ethics Committee. Written informed consent to participate in this study was provided by the participants' legal guardian/next of kin.

## Author contributions

YW collected data of Chinese migrant families and took the lead in writing the manuscript. VWT and SD collected data of African migrants, analyzed the data, provided critical feedbacks, and helped to shape the paper. All authors contributed to the article and approved the submitted version.

## Footnotes

^1^The Higher School Certificate (HSC) is the culmination of the school career and the highest educational award you can achieve at secondary school in New South Wales.
